# Requalification of patients with severe asthma for biological therapy—Practical ‘ReQuaBi’ rate decision scheme based on the analytical model

**DOI:** 10.1002/clt2.70059

**Published:** 2025-05-09

**Authors:** Alicja Majos, Anna Ben Drissi, Maciej Kupczyk, Michał Panek

**Affiliations:** ^1^ Department of General and Transplant Surgery Medical University of Lodz Łódź Poland; ^2^ Department of Internal Medicine, Asthma and Allergy Medical University of Lodz Łódź Poland; ^3^ Polish Society of Allergology Coalition for Asthma Treatment Łódź Poland

**Keywords:** adult, asthma, asthma management, biologics, GINA, requalification

## Abstract

**Background:**

Patients with severe asthma experience decreased quality of life due to fixed airway obstruction, hospitalisations and potential fatalities. However, to date, the requalification of severe asthma patients eligible for biological therapy in daily clinical practice remains unstudied.

**Objective:**

The aim of the study was to prepare a universal decision‐making algorithm for requalifying patients for biological therapy based on available clinical data obtained from a leading reference centre in Poland.

**Methods:**

All severe asthma patients treated with biologics since 2013 at the Internal Medicine, Asthma and Allergy Department (Medical University of Lodz, Poland), were analysed. The analysis included demographic (age, sex), pre‐treatment (reported at qualification: oral glucocorticosteroids use, total IgE serum level, peripheral blood eosinophilia, co‐morbidities: atopic dermatitis, chronic allergic rhinitis or sinusitis) and treatment‐related data (treatment time, current treatment status, reason for early termination of therapy, year of discontinuation, rediagnostics, requalification).

**Results:**

Rediagnostics were performed in only 4.76% of all requalifications. The following additional data were used to requalify patients: blood eosinophilia (*n* = 63; 100.00% of requalifications), atopic comorbidities (*n* = 30; 47.62%) and total IgE serum level (*n* = 8; 12.70%). Kaplan–Meier curve analysis of all source data revealed the longevity of maintenance as follows: the highest for mepolizumab, then omalizumab, benralizumab, dupilumab and tezepelumab (*p* = 0.016). Based on the results, requalification model ‘ReQuaBi’, was constructed.

**Conclusion:**

The most important criteria for selecting a biological agent in requalification are peripheral blood eosinophilia, followed by comorbidities and IgE levels. In most cases, extensive additional re‐diagnosis may not be necessary.

## 1 INTRODUCTION

Severe asthma represents a challenge for patients, clinicians and health care systems. Patients experience decreased quality of life, fixed airway obstruction, hospitalisations, and potential fatalities.^1^, ^2^ Despite optimal treatment, some patients with severe asthma (3.7%) still require high intensity treatment associated with significant risk of long term aside effects, and have poor symptom control.^3^, ^4^, ^5^ Biologics can decrease disease progression, help improve symptom control and may lead to asthma remission.

Patients with severe asthma can receive biological treatment comprising anti‐IgE, anti‐IL‐5/anti‐IL5R, anti‐IL‐4R and anti‐TSLP; the allocation criteria is based on an algorithm comprising phenotyping, endotyping including several biomarkers and the presence of comorbidities as presented by GINA 2024.^1^, ^3^ However, despite the optimal qualification for biological therapy, some patients still do not respond to treatment. In such cases, it is recommended to reassess the differential diagnosis as well as adherence, coexisting conditions, phenotypes and biomarkers, stop ineffective treatment and consider switching to a different biological agent.^2^, ^6^, ^7^, ^8^

Several factors which may predict good asthma response to biologics have been proposed. Conversely, the following criteria have been found to be indicative of a sub‐optimal response: (a) decrease in FEV1 ≥ 25% and 200 mL from pretreatment value, (b) increase in MCS (maintenance corticosteroid dose) and (c) increase in ACQ by ≥ 0.5 from pretreatment value. On this basis, as many as 30% of patients qualified for biological therapy have been estimated to have a sub‐optimal response.^3^, ^8^, ^9^, ^10^

To date, no study has described the principles for requalification of patients with severe asthma who are eligible for biological therapy in clinical practice. Therefore, the aim of the study was to propose a universal decision‐making algorithm for requalifying patients for biological therapy with a second drug based on their exclusion from previous treatment. The algorithm was prepared by analysing clinical data on re‐qualification for biological therapy collected from the leading asthma reference centre in Poland.

## 2 MATERIALS AND METHODS

### 2.1 Definitions

The following reasons for discontinuation were used in the study: (1) treatment failure (treatment response below good according to GETE scale; frequency of exacerbations higher than before treatment, no improvement in ACQ or AQLQ questionnaire results) OR treatment complications (joint or muscle pain; eosinophilic pneumonia or hypereosinophilia); (2) patient death and (3) other reasons.

Rediagnostics was defined as the performance of at least one diagnostic test before requalification, aiming to exclude possible reasons other than severe asthma for the failure of the first biological course: chronic obstructive pulmonary disease, hypereosinophilic conditions etc. The extensive potential diagnostic workout included inter alia spirometry with bronchodilator test, chest imaging, haematologist consultation or bronchoscopy with histopathological examination of bronchial or bronchoalveolar lavage samples. Requalification was defined as starting a new biological drug after the failure of the initial one. Chronic oral corticosteroid (OCS) use was referred to daily use during the last 6 months before qualification. Each qualification was considered as one record.

### 2.2 Study design

The analysis included all patients treated with biologics since 2013 at the Department of Internal Medicine, Asthma and Allergy (Medical University of Lodz, Poland), the leading reference centre for the management of severe asthma in Poland. The following sets of data were collected: Demographic data (age, sex); pre‐treatment data reported at qualification (oral glucocorticosteroid use, total IgE serum level, peripheral blood eosinophilia, co‐morbidities: atopic dermatitis, chronic allergic rhinitis or sinusitis) and treatment‐related data (qualification and discontinuation dates, treatment time, current treatment status, reason for termination of biologic, re‐diagnostics, requalification).

Only adult patients were included, that is, aged over 18 years. The analysis included courses starting before 20.06.2024 and ending before 20.10.2024. The dates of the courses were determined based on data concerning reimbursement for biologics obtained from the NFZ (national health fund). The first dates of enrolment for treatment were as follows: omalizumab—05.04.2013; mepolizumab—08.03.2018; benralizumab—18.11.2019; dupilumab—05.12.2022; tezepelumab—14.05.2024. In addition, the requalification data were also checked for patients who failed to complete treatment with the second drug due to ineffectiveness or complications.

**FIGURE 1:**
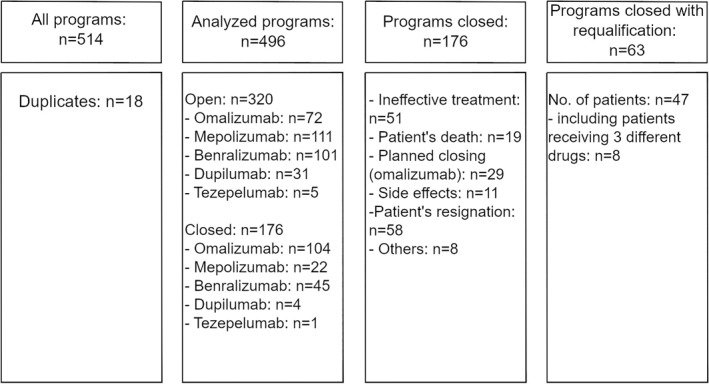
Study design.

The study design is presented in Figure 1. For each patient, the analysis included mean OCS use per day for 12 months before biological treatment and qualification data: that is, total serum IgE but not peripheral eosinophilia for omalizumab treatment, and peripheral eosinophilia but not total serum IgE for mepolizumab, benralizumab and dupilumab treatment. All available medical records were also recorded: rediagnostic data, comorbidities reported, peripheral blood eosinophilia level and total serum IgE level. The length of the treatment is presented in full months.

### 2.3 Statistical analysis

Statistical analysis was conducted with Statistica 13 PL. Figures were created using Statistica 13 PL or Gimp 2.10.38. Nominal variables were compared using Pearson's chi‐square test, while continuous variables were analysed with the Kruskal‐Wallis test for multiple comparisons. Adherence to treatment was estimated through the Kaplan–Meier method with a log‐rank test equivalent for multiple‐group comparisons. Complete observations were defined as termination due to the treatment failure or treatment complications. Statistical significance was defined as *p* < 0.05 for all tests.

### 2.4 ‘ReQuaBi’ analytical model

The requalification model, proposed in this publication, was created using the following data: (1) effectiveness data from all source data analysis; (2) safety data from all programme analysis; (3) qualification data; (4) continuation/discontinuation analysis of requalification cases and (5) validation by calculating specificity/sensitivity and comparing with literature values.

### 2.5 Opinion of the bioethics committee at the medical university of Lodz, Poland

The study does not require Bioethics Committee approval, as indicated by the Medical University of Lodz Bioethics Committee notification (Number RNN/58/25/KE of 11.02.2025).

## 3 RESULTS

### 3.1 All source data analysis

In total, 496 therapies were included in the analysis: of these, *n* = 320 were ongoing, and *n* = 176 terminated. Women participated in 61.69% of all therapeutic programs (*n* = 306) and were in comparable age to the men (median age of woman: 59.0 years; IQR 48.0–68.0 vs. median age in men: 56.0; IQR 48.0–68.0; *p* = 0.355). Potential reasons for treatment discontinuation are presented in Table 1.

**TABLE 1:** Potential reasons for treatment discontinuation (number and % of discontinued therapies) according to the drug used.

Drug used		Ineffectiveness	Side effects	Patient's resignation	Patient's death	Other	Planned discontinuation
Mepolizumab	*n*	7	0	10	3	2	0
	%	31.82%	0.00%	45.45%	13.64%	9.09%	0.00%
Omalizumab	*n*	19	6	33	8	9	29
	%	18.27%	5.77%	31.73%	7.69%	8.65%	27.88%
Benralizumab	*n*	22	0	15	8	0	0
	%	48.89%	0.00%	33.33%	17.78%	0.00%	0.00%
Dupilumab	*n*	2	2	0	0	0	0
	%	50.00%	50.00%	0.00%	0.00%	0.00%	0.00%
Tezepelumab	*n*	1	0	0	0	0	0
	%	100.00%	0.00%	0.00%	0.00%	0.00%	0.00%
Sum	*n*	51	8	58	19	11	29
	%	28.98%	4.55%	32.95%	10.80%	6.25%	16.48%

The ineffectiveness rate was low for benralizumab (*n* = 22; 48.89%), mepolizumab (*n* = 7; 21.82%) and omalizumab (*n* = 19; 18.27%). Reported side effects included bone or joint pain (*n* = 5), and diarrhoea (*n* = 1) for omalizumab, and hypereosinophilia (*n* = 2) for dupilumab. Similar resignation rates were noted for mepolizumab, omalizumab and benralizumab (21.73%–45.45%). Other reasons for early termination for omalizumab treatment included malignant neoplasia treatment (pulmonary cancer *n* = 1, breast cancer *n* = 1), worsening of ankylosing spondylitis with good control of asthma (*n* = 1), need for anti‐rabies treatment (*n* = 1), lack of compliance (*n* = 1), acute cardiological condition (*n* = 1) and physician's decision about planned modification of treatment (*n* = 3). In two cases, lack of compliance led to resignation from mepolizumab (*n* = 2).

**FIGURE 2:**
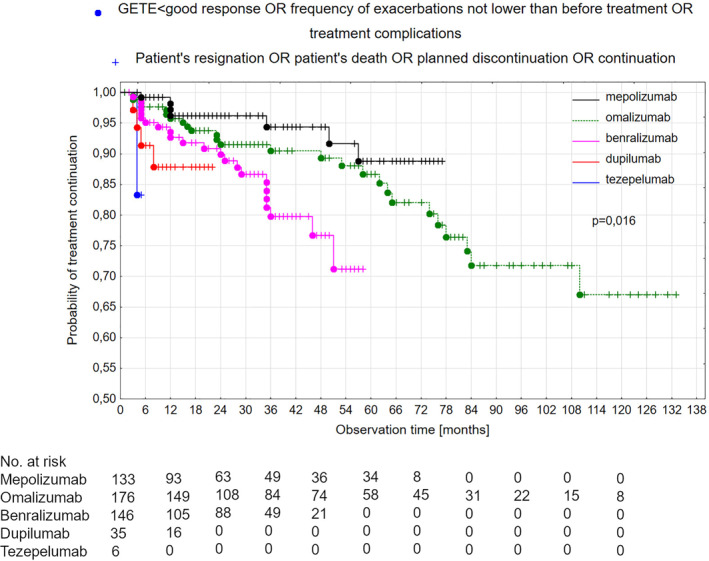
Survival curves for analysed therapeutic programs (*n* = 496). Curve separation achieved statistical significance (*p* = 0.02).

Kaplan–Meier curves demonstrating treatment continuation are presented in Figure 2. Longer maintenance was observed for mepolizumab compared with omalizumab, benralizumab, dupilumab or tezepelumab. Separation of the curves is clearly visible and statistically significant (*p* = 0.016).

To analyse the reasons for treatment termination and to make them more comparable, the following ratios of events were calculated: closure due to ineffectiveness of treatment or treatment complications/100 programs/year × 10 and closure due to death/100 programs/year × 10 (Table S1). The dates of each death are presented in Table S2 together with the length of treatment. The following median time of treatment until patient death was noted: 24.0 months for omalizumab, 21.0 months for mepolizumab and 18.0 months for benralizumab.

### 3.2 Requalification data

The highest number of requalifications was needed in patients treated with omalizumab as the initial biological agent (*n* = 43; 53.97%), followed by benralizumab (*n* = 15; 23.81%), mepolizumab (*n* = 11; 17.46%) and dupilumab (*n* = 3; 4.75%). The most frequently selected second drug was benralizumab (*n* = 24; 38.75% of all requalifications), followed by dupilumab (*n* = 20; 31.75%), mepolizumab (*n* = 11; 17.46%), tezepelumab (*n* = 6; 9.52%), and omalizumab (*n* = 2; 3.17%). Further data regarding the direction of requalification are presented in Table 2.

**TABLE 2:** Summary of the direction of requalifications.

Initial drug	Drug used after requalification; *n* (%)				
	Omalizumab	Mepolizumab	Benralizumab	Dupilumab	Tezepelumab
Omalizumab	–	5 (14.71%)	17 (50.00%)	11 (32.35%)	1 (2.94%)
Mepolizumab	0 (0.00%)	–	7 (63.64%)	3 (27.27%)	1 (9.09%)
Benralizumab	1 (6.67%)	5 (33.33%)	–	6 (40.00%)	3 (20.00%)
Dupilumab	1 (33.33%)	1 (33.33%)	0 (0.00%)	–	1 (33.33%)
Tezepelumab	0 (0.00%)	0 (0.00%)	0 (0.00%)	0 (0.00%)	–

*Note*: Data expressed as: number of therapies (percentage of requalifications from the initial drug).

*p* = 0.002.

**FIGURE 3:**
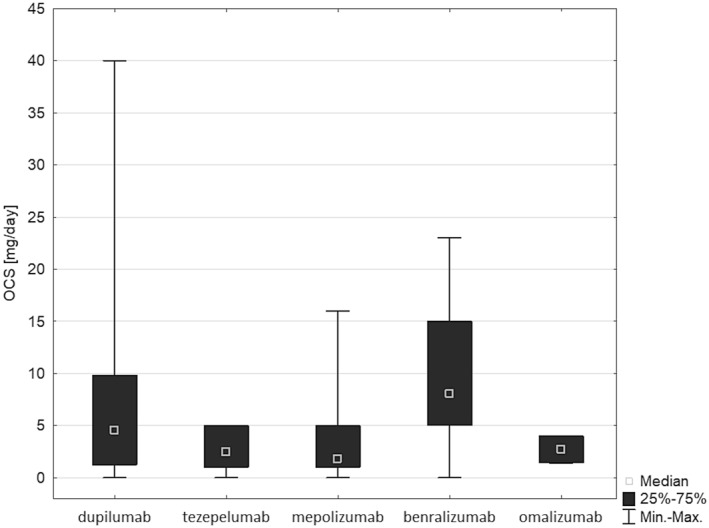
Oral corticosteroid use (OCS [mg/day]) during a year before start of the biological treatment, recalculated to prednisone use in milligrammes per day as reported in therapeutic programs, according to biological drug applied; *p* = 0.03 and p post hoc ≥0.05 (requalified patients and biologicals after requalification).

The influence of oral corticosteroid use during the previous year on the effectiveness of biological treatment was also analysed. Although some differences are noticeable between first choice therapies (Figure 3), they are not statistically significant (*p* > 0.05). The effect of chronic OCS therapy status on requalification results is presented in Figure 1. It is worth noting that benralizumab is preferred over mepolizumab when requalifying from omalizumab.

Extensive additional examinations during rediagnostics were performed only in 4.76% of all requalifications: two cases before requalification to benralizumab and one case before requalification to dupilumab. The following additional data were used to requalify patients: blood eosinophilia (*n* = 63; 100.00% of requalifications), comorbidities (*n* = 30; 47.62%) of which dupilumab qualifications constituted *n* = 13 (65%), and total IgE serum level (*n* = 8; 12.70%), tested in two (100.00%) omalizumab qualification cases (Table S3). At the time of the current study, *n* = 52 (82.54%) therapies chosen after requalification were ongoing. Only 11 second‐choice therapies were terminated including benralizumab (*n* = 6; 9.52% of all requalifications), dupilumab (*n* = 3; 4.76%) and mepolizumab (*n* = 2; 3.17%).

Further data regarding the discontinuation of the second‐choice drug due to its ineffectiveness or side effects are given in Table S4. This includes six patients requalified for benralizumab (26.09% of all benralizumab requalifications) and three requalified for dupilumab (15.00% of all dupilumab requalifications). Among these, treatment complications were responsible for discontinuation in one patient on dupilumab, diagnosed with hypereosinophilia; the subject started therapy with blood eosinophilia 630 G/l, without reported atopic conditions and with unknown total serum IgE level.

### 3.3 Requalification scheme

**FIGURE 4:**
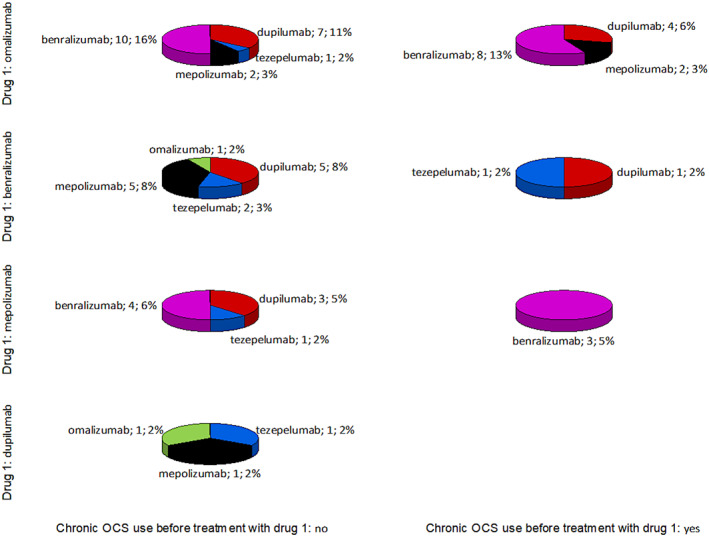
Pie charts present biological drugs chosen after requalification in patients on or without oral corticosteroids chronic therapy. The results are presented as name of the second drug; number of cases and percentage of all requalifications.

**FIGURE 5:**
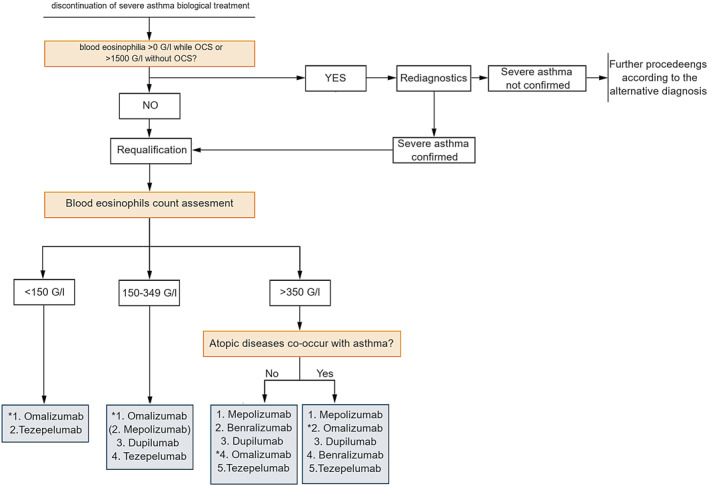
Proposition of a requalification scheme based on the current study data and literature. The term ‘discontinuation’ refers to discontinuation of biological treatment in patients who had to stop initial severe asthma biological treatment and need to start an alternative one. Numbers: 1–5 refer to proposed order of therapy selection. * for patients who can benefit from therapeutic use of omalizumab (allergic asthma, perennial allergen and tIgE > 30). Atopic diseases co‐occurring with asthma: allergic rhinitis/chronic sinusitis with nasal polyps, atopic dermatitis; ()—mepolizumab use in Polish patients with blood eosinophilia 150–340 G/l is possible since year 2025; IMPORTANT: as currently there is no evidence that tezepelumab can lead to OCS withdrawal in patients chronically using OCS; so we recommend its use only for patients who are not OCS‐dependent. OCS, oral corticosteroids.

A requalification algorithm was prepared based on our present and literature data (Figure 4). To validate this model, all requalifications from the studied Department were compared with the algorithm proposed by Brussels et al.,^2^ based on the continuation or discontinuation status of the therapy (Figure 5). It was found that *n* = 63 (100.00%) allocations to therapy were consistent with Brussels et al.; this also applies to all nine (100.00%) cases of requalifications resulting in discontinuation due to treatment failure or complications. In our algorithm, the numbers are consecutively: *n* = 29 (46.03%) and *n* = 2 (22.22%). Therefore, when detecting possible treatment failure after requalification, the Brussels algorithm demonstrated 0.00% sensitivity (0.00%–33.63%) and 100.00% specificity (93.40%–100.00%), compared to 77.78% (39.99%–97.19%) and 53.70% (39.61%–67.38%) for the proposed algorithm.

## 4 DISCUSSION

Although severe asthma represents a diagnostic and therapeutic challenge,^3^ a body of research indicates that biological treatment may play an important role in its management. Several guidelines on the first choice of biologics in asthma have been proposed^1^, ^2^, ^3^; however, few data exist on the lack of response and the process of requalification of patients for a second biological agent.^1^, ^11^, ^12^ Our paper represents one of the first reports on real‐life data on the requalification of patients for biological therapy in severe asthma. This original approach to the topic is well suited to everyday clinical practice. Our data has been used to prepare a practical algorithm for requalification to biological therapy in severe asthma.

Our study of a large cohort of patients whose biological therapy had been terminated found that different biologicals are characterised by varying degrees of real‐world effectiveness and safety. Requalification decisions are not made based on chronic OCS use, cumulative OCS dose, comorbidities, drug characteristics, or rediagnosis results, that is, not on a holistic picture of the patient, but only on the parameters necessary to administer a given Ab. The discrepancies between treatment results and the frequency of use of anti‐IL5 drugs are particularly noteworthy (Table S1, Figure S1).^7^, ^8^, ^13^

Our analysis included several criteria and medical data used to select the second course of biological therapy if the patient did not respond to first‐line treatment. It is clear that this decision is not generally made based on extensive additional diagnostic workup. Our analysis of all available medical records indicated that the most important criterion for choosing a second biological agent is the level of peripheral blood eosinophils during the process of requalification, followed by the presence of comorbidities and the level of total IgE. No other medical data influenced the choice of biological agent during the requalification process, or no such data was reported. Interestingly, it seems unlikely that the use of OCS before requalification represents a key factor in the qualification.

The analysis also included an assessment of the effectiveness of the second‐line biological samples selected after requalification. The highest effectiveness based on the proposed requalification criteria (*viz*. blood eosinophil level, presence of comorbidities and serum IgE level) was found for omalizumab (100.00%) and tezepelumab (100.00%) followed by dupilumab, mepolizumab and benralizumab. However, this analysis was limited by the short observation time (tezepelumab) and small group of requalified patients (omalizumab). The requalification criteria was found to be least effective for benralizumab (24.00%), as indicated by the ‘treatment ended’ (all reasons) criterion.

Treatment compliance curves showed clear separation, confirming that the tested biologicals perform differently in real‐life management of severe asthma. Interestingly, benralizumab was found to perform significantly worse than mepolizumab, despite being usually allocated to the same category.^2^, ^3^ However, the relatively high median pre‐treatment OCS dose noted before requalification to benralizumab suggests that patients in this group could be the most difficult to treat, which can influence the effectiveness and death rate data. However, requalification to benralizumab may have driven the desire to use a new therapeutic option, and its use may have been discouraged during the COVID‐19 pandemic. Based on our results, especially the treatment results and Kaplan–Meier curve analysis, we propose that in contrast to previous schemes, anti‐IL5 and anti‐IL5R therapy should be considered separately.^2^, ^3^

Our analysis allowed the development of the ‘ReQuaBi’ algorithm, which may help in guiding decisions regarding second‐choice therapy in severe asthma in every‐day clinical practice. The proposed algorithm has been validated against other available qualification schemes and takes into account all data available to specialists in their daily routine. It may assist the decision making in patients with severe asthma who fulfil the criteria for several biological agents, additionally guiding a more detailed retrospective analysis of real‐world data. Although innovative, the proposed requalification scheme is consistent with GINA, where the add‐on biological drug scheme proposes the order of selection based on availability and eligibility criteria.^2^

### 4.1 Study limitations

The main limitation of the study is its retrospective nature which would affect data quality and availability. Also, the analysis did not include any information about the reasons for not requalifying patients whose first‐choice treatment was terminated due to ineffectiveness and were not restarted with another mAb. Also, some differences between clinical control and safety may result from the length of follow‐up in therapy rather than the effectiveness and safety of a specific mAb (dupilumab, tezepelumab); this may result from the availability of the given biological agent due to the date of registration and reimbursement.

## 5 CONCLUSIONS

The most important criterion for selecting a biological agent during requalification of severe asthma patients is peripheral blood eosinophilia, followed by comorbidities and the level of IgE; however, chronic OCS use does not represent a significant factor. Most strikingly, no extensive additional re‐diagnostic workup was necessary in most cases. Our proposed practical algorithm may optimise the allocation of patients to treatment with second‐line biological agents as part of everyday clinical practice. Such a system will alleviate the burden of severe asthma for patients, their families and health care systems.

## AUTHOR CONTRIBUTIONS

**Alicja Majos**: Methodology; writing—original draft; visualization; validation; formal analysis. **Anna Ben Drissi**: Project administration. **Maciej Kupczyk**: Validation; formal analysis; supervision. **Michał Panek**: Conceptualization; investigation; funding acquisition; writing—original draft; writing—review and editing; software; project administration; data curation; supervision; resources. **Michal Panek**: Investigation; funding acquisition; writing—review and editing; project administration; software; data curation; supervision; resources.

## CONFLICT OF INTEREST STATEMENT

The authors declare no conflicts of interest.

## Supporting information

Supporting Information S1

Figure S1

## Data Availability

Data sharing is not applicable to this article as no new data were created or analyzed in this study.
