# Supplementary material for: Requalification of patients with severe asthma for biological therapy—Practical ‘ReQuaBi’ rate decision scheme based on the analytical model
Source: Clin Transl Allergy. 2025 May 9;15(5):e70059. doi: 10.1002/clt2.70059 (PMC12063523; doi:10.1002/clt2.70059)
Supplement: Supplementary file 1 — Supporting Information S1 [file CLT2-15-e70059-s001.docx]

**Material and Methods**

*Polish National Program of biological treatment of severe asthma*

Therapeutic program is a special form of treatment reimbursement. In Polish National Program of Severe Asthma Treatment, biological drug use is free of payment but has some limitations – strictly described qualification criteria and restrictions considering treatment continuation. Also, drug availability is limited in time as usually drug registration proceeds Program novelization. Program treatment with omalizumab started in 2013; mepolizumab – 2018; benralizumab – 2019; dupilumab – 2022; Tezepelumab – 2024. Also, qualification criteria for a specific biological were updated – mepolizumab initially was available for patients with blood eosinophilia >=0,35 G/l, similarly to benralizumab, in 2025 there will be also possible to use it when blood eosinophilia equals 0,15-0,5 G/l (like dupilumab). Omalizumab use requires total serum IgE >30 kU/l and allergic asthma; patients with too high body weight in relation to total IgE are excluded from the program (according to the registration record). Tezepelumab, although already published data contraindicating its use in OCS-dependent patients [11] is allowed in every severe asthma patient. There are differences in patient’s age allowing participation, but all listed drugs are possible to use in adults since their first mentioning in Program.

Supplementary Table 1. Summary of information about terminated therapies. A - Number of terminated therapies due to ineffectiveness of treatment or treatment complications; B - Number of terminated therapies due to patient’s death; C – number of all analysed therapies, D – observation time. ^1^ – approximate data, as the real observation time is not yet a full year.

| Drug used | A | B | C | D | A/C | A/100 programs/year*10 | B/C | B/100 programs/year*10 |
| --- | --- | --- | --- | --- | --- | --- | --- | --- |
| Mepolizumab | 7 | 3 | 133 | 6 | 2,26% | 8,77 | 2,26% | 3,75 |
| Omalizumab | 26 | 8 | 176 | 11 | 14,77% | 13,43 | 4,54% | 4,13 |
| Benralizumab | 23 | 8 | 146 | 4 | 15,75% | 39,38 | 5,48% | 13,67 |
| Dupilumab | 4 | 0 | 35 | 2 | 11,43% | 57,14 | 0% | 0 |
| Tezepelumab^1^ | 1 | 0 | 6 | 1 | 16,67% | 166,67 | 0% | 0 |

Supplementary table 2. Patients’ death/s dates with length of therapy with biologics.

| Biological drug used | No. | Death date | Number of months in program |
| --- | --- | --- | --- |
| omalizumab | 1 | 26.03.2015 | 17 |
|  | 2 | 15.11.2016 | 37 |
|  | 3 | 22.10.2018 | 24 |
|  | 4 | 01.11.2018 | 48 |
|  | 5 | 29.11.2018 | 10 |
|  | 6 | 10.07.2019 | 37 |
|  | 7 | 21.05.2021 | 15 |
|  | 8 | 01.07.2023 | 86 |
| mepolizumab | 1 | 08.11.2021 | 8 |
|  | 2 | 08.11.2021 | 30 |
|  | 3 | 09.07.2022 | 21 |
| benralizumab | 1 | 25.12.2020 | 2 |
|  | 2 | 06.10.2022 | 14 |
|  | 3 | 26.12.2022 | 33 |
|  | 4 | 17.01.2023 | 2 |
|  | 5 | 13.04.2023 | 18 |
|  | 6 | 21.10.2023 | 27 |
|  | 7 | 27.01.2024 | 41 |
|  | 8 | 05.03.2024 | 38 |

Supplementary Table 3. The availability of requalifications data; Available/not available – information available or not in patient’s medical records. Comorbidity: unsure – in patient’s medical records there is no available information about comorbidities as atopic dermatitis, allergic rhinitis, chronic sinusitis or no test results allowing for exclusion of such diseases. Total IgE serum level: unsure – in cases of requalification from omalizumab without retesting tIgE level.

| Drug used after requalification | Blood eosinophilia | Comorbidity | | Total IgE serum level | | |
| --- | --- | --- | --- | --- | --- | --- |
|  | Available | Available | Unsure | Available | Unsure | Not available |
| Omalizumab | N=2; 100,00% | N=1, 50,00% | N=1; 50,00% | N=2; 100,00% | - | - |
| Mepolizumab | N=11; 100,00% | N=2; 18,18% | N=9; 81,82% | - | N=5; 45,45% | N=6; 54,55% |
| Benralizumab | N=24; 100,00% | N=11; 45,83% | N=13; 54,17% | - | N=17; 70,83% | N=7; 29,17% |
| Dupilumab | N=20; 100,00% | N=13; 65,00% | N=7; 35,00% | N=5; 25,00% | N=6; 30,00% | N=9; 45,00% |
| Tezepelumab | N=6; 100,00% | N=3; 50,00% | N=3; 50,00% | N=1; 16,67% | - | N=5; 83,33% |
| Total | N=63; 100,00% | N=30; 47,62% | N=33; 52,38% | N=8; 12,70% | N=28; 44,44% | N=27; 42,86% |

Supplementary Table 4. Summary of ineffective requalifications (second therapy terminated due to the ineffective treatment or its complication). EOS – peripheral blood eosinophilia; comorbidity – refers to atopic conditions: all patients stated YES in this table presented allergic rhinitis or chronic sinusitis; * hypereosinophilia; () – therapy not analysed in survival analysis due to the starting date after 06.2024

| No | Initial drug | Requalification data | | | Second drug | Third drug |
| --- | --- | --- | --- | --- | --- | --- |
|  |  | EOS [G/l] | tIgE | Comorbidity |  |  |
| 1. | Omalizumab | 350 | - | NO | Benralizumab | Dupilumab |
| 2. | Omalizumab | 360 | - | YES | Benralizumab | Dupilumab |
| 3. | Omalizumab | 420 | - | YES | Benralizumab | (Mepolizumab) |
| 4. | Omalizumab | 500 | - | NO | Benralizumab | Omalizumab |
| 5. | Omalizumab | 620 | - | NO | Benralizumab | (Tezepelumab) |
| 6. | Omalizumab | 1310 | - | NO | Benralizumab | Mepolizumab |
| 7. | Omalizumab | 170 | ≥30 kU/l | YES | Dupilumab | (Tezepelumab) |
| 8. | Omalizumab | 350 | ≥30 kU/l | YES | Dupilumab | Omalizumab |
| 9. | Benralizumab | 630 | - | NO | Dupilumab* | Mepolizumab |
