# Supplementary material for: Requalification of patients with severe asthma for biological therapy—Practical ‘ReQuaBi’ rate decision scheme based on the analytical model
Source: Clin Transl Allergy. 2025 May 9;15(5):e70059. doi: 10.1002/clt2.70059 (PMC12063523; doi:10.1002/clt2.70059)
Supplement: Supplementary file 2 — Figure S1 [file CLT2-15-e70059-s002.docx]

Supplementary Figure 1. Pie charts present biological drugs chosen after requalification in patients with or without oral corticosteroids chronic therapy (OCS). The results are presented as: name of the second drug; number of cases; percentage of all requalifications.
